# Sexual dimorphism in the relationship between BMI and recent suicidal attempts in first-episode drug-naïve patients with major depressive disorder

**DOI:** 10.1186/s40779-024-00572-1

**Published:** 2024-09-26

**Authors:** Ze-Zhi Li, Yu-Ping Chen, Xiao-Cui Zang, Denise Zheng, Xiao-E Lang, Yong-Jie Zhou, Feng-Chun Wu, Xiang-Yang Zhang

**Affiliations:** 1 Department of Nutritional and Metabolic Psychiatry, the Affiliated Brain Hospital, Guangzhou Medical University, Guangzhou, 510370 China; 2 Guangdong Engineering Technology Research Center for Translational Medicine, Guangzhou, 510370 China; 3 Key Laboratory of Neurogenetics and Channelopathies of Guangdong Province, Ministry of Education of China, Guangzhou Medical University, Guangzhou, 510145 China; 4 Jiangsu Key Laboratory of Neurodegeneration, Nanjing Medical University, Nanjing, 211166 China; 5 Qingdao Mental Health Center, Qingdao, 266034 Shandong China; 6 McGovern Medical School, the University of Texas Health Science Center at Houston, Houston, TX 77030 USA; 7 Department of Psychiatry, the First Clinical Medical College, Shanxi Medical University, Taiyuan, 030606 China; 8 Shenzhen Kangning Hospital, Shenzhen, 518000 Guangdong China; 9 Department of Psychiatry, the Affiliated Brain Hospital of Guangzhou Medical University, Guangzhou, 510370 China; 10 CAS Key Laboratory of Mental Health, Institute of Psychology, Chinese Academy of Sciences, Beijing, 100101 China

**Keywords:** Sexual dimorphism, Body mass index, Suicidal attempts, Major depressive disorder

Dear Editor,

Major depressive disorder (MDD) is associated with a high rate of suicide attempts (SA). Previous reports have identified risk factors for SA in MDD patients, including sex and metabolic disorders [1]. However, to our knowledge, the impact of sex differences on the association between body mass index (BMI) status and SA in patients with MDD has not been investigated. This study aimed to investigate: 1) the sex difference in the prevalence of recent SA in Chinese drug-naïve first-episode (DNFE) MDD patients, and 2) the effect of sex difference on the relationship between higher BMI (overweight or obese) and recent SA. Patients were recruited from the First Clinical Medical College of Shanxi Medical University from January 2015 to December 2017. All patients met the inclusion and exclusion criteria described in previous studies [2].

Patients were interviewed by 2 independent psychiatrists using the Structure Clinical Interview for DSM-IV (SCID-I/P). Depressive symptoms were evaluated by a 17-item Hamilton Rating Scale for Depression (HAMD-17), and anxiety symptoms were assessed with the Hamilton Anxiety Rating Scale (HAMA). The inter-rater correlation coefficients for the assessments exceeded 0.8. In this study, a suicide attempt was defined as a deliberate self-destructive behavior with at least some intent to die but did not result in death. SA was assessed within 1 month, including specific dates, times, and methods used. A 2 × 2 analysis of covariance (ANCOVA) was performed to analyze the interaction between sex and SA, along with univariate regression analysis and multivariate logistic regression was performed.

A total of 1718 eligible outpatients (588 males and 1130 females) were enrolled in this study. Within 1 month of the assessment, 235 MDD patients (13.7%) exhibited SA. Among the 1718 MDD patients, 676 patients (39.4%) were classified as overweight, 3 patients (0.2%) as obese, and 10 patients (0.6%) as underweight.

After analyzing the data presented in Table 1, it was found that male patients were younger compared with female patients (*P* < 0.001). Furthermore, the prevalence of recent SA was observed to be higher in female patients as opposed to male patients (*P* = 0.03, *OR* = 1.40, 95% CI 1.03 − 1.90). The demographic and clinical characteristics between SA and non-SA patients were outlined in Additional file 1: Table S1. Additionally, an interactive effect on BMI level between sex and SA was identified as demonstrated in Additional file 1: Table S2. Specifically, after adjusting for age and age of onset, it was noted that female MDD patients with SA exhibited a higher BMI compared with those without SA. Moreover, the overweight rate remained elevated in female patients with SA when compared with those without SA (*P* = 0.006, *OR* = 1.58, 95% CI 1.14 − 2.19). As shown in Additional file 1: Table S3, both BMI and overweight were found to be correlated with SA in female patients (*P* < 0.01). The findings are further highlighted by the results presented in Additional file l: Table S4 (BMI, *P* = 0.005) and Additional file l: Table S5 (overweight, *P* = 0.016).

**Table 1:** Demographic and clinical characteristics of male and female patients with major depressive disorder (MDD)

Variable	Male (*n* = 588)	Female (*n* = 1130)	t/χ^2^	*P*
Age (year)	33.1 ± 12.2	35.8 ± 12.5	4.22	< 0.001
Age of onset (year)	32.9 ± 12.1	35.6 ± 12.3	4.26	< 0.001
HAMD-17 score	30.3 ± 3.0	30.3 ± 2.9	0.18	0.86
HAMA score	20.7 ± 3.4	20.9 ± 3.5	1.20	0.23
BMI (kg/m^2^)	24.4 ± 2.0	24.4 ± 1.9	0.62	0.54
Overweight [*n* (%)]	239 (40.6)	440 (38.9)	0.47	0.49
SA [*n* (%)]	65 (11.1)	171 (15.1)	5.43	0.02

*HAMD-17* 17-item Hamilton Rating Scale for Depression, *HAMA* Hamilton Anxiety Rating Scale, *BMI* body mass index, *SA* suicide attempts

We observed a higher prevalence of recent SA in female patients with MDD compared with male patients. Female MDD patients with SA also exhibited elevated levels of BMI and higher rates of overweight, in contrast to those without such attempts. Additionally, we found a positive association between BMI levels and overweight only in female patients with SA. The underlying mechanism of this association among female patients is unclear; however, several potential explanations exist. Firstly, previous research has indicated that high BMI is linked to poor quality of life, which subsequently increases comorbidities of depression and heightened suicide risk [3]. Notably, this relationship is influenced by sex and is more pronounced among women than men. Secondly, the connection between BMI and SA in women may be more complex than that in men due to factors such as weight dissatisfaction, stigma, and discrimination playing a more prominent role for women. Individuals who are obese often face stigmatization leading to adverse psychological effects [4]. Thirdly, sex differences in physical activity could be a plausible explanation as increased physical activity is associated with reduced SA risk [5]. While all these factors may elucidate the sex difference in the association between BMI and SA among individuals with MDD, further investigation into the exact mechanisms is warranted.

Our findings suggest that clinicians should take into consideration the impact of sexual dimorphism on the relationship between BMI and SA in MDD patients.

## Supplementary Information

**Additional file 1: Table S1 **Demographic and clinical characteristics of major depressive disorder (MDD) patients with SA and non-SA. **Table S2 **Demographic and clinical characteristics in SA and non-SA patients categorized by sex. **Table S3 **Association between each variable and SA in major depressive disorder (MDD) by categorized by sex using univariate logistic regression. **Table S4 **Association between BMI and SA in major depressive disorder (MDD) by categorized by sex using multivariate logistic regression. **Table S5 **Association between overweight and SA in major depressive disorder (MDD) by categorized by sex using multivariate logistic regression.

## Data Availability

Data will be available from the corresponding author upon reasonable request.

## Abbreviations

BMI: Body mass index
MDD: Major depressive disorder
SA: Suicide attempts
