# Supplementary material for: Sexual dimorphism in the relationship between BMI and recent suicidal attempts in first-episode drug-naïve patients with major depressive disorder
Source: Mil Med Res. 2024 Sep 26;11:66. doi: 10.1186/s40779-024-00572-1 (PMC11425971; doi:10.1186/s40779-024-00572-1)
Supplement: Supplementary file 1 — Additional file 1: Table S1 Demographic and clinical characteristics of major depressive disorder (MDD) patients with SA and non-SA. Table S2 Demographic and clinical characteristics in SA and non-SA patients categorized by sex. Table S3 Association between each variable and SA in major depressive disorder (MDD) by categorized by sex using univariate logistic regression. Table S4 Association between BMI and SA in major depressive disorder (MDD) by categorized by sex using multivariate logistic regression. Table S5 Association between overweight and SA in major depressive disorder (MDD) by categorized by sex using multivariate logistic regression. [file 40779_2024_572_MOESM1_ESM.pdf]

Table S1 Demographic and clinical characteristics of major depressive disorder (MDD) patients with SA and non-SA

**Variable**	**SA (*n* = 236)**	**Non-SA (*n* = 1482)**	t/χ^2	***P***
Age (year)	36.5 ± 12.6	34.6 ± 12.4	2.13	0.03
Age of onset (year)	36.3 ± 12.5	34.4 ± 12.3	2.15	0.03
Sex [male, *n* (%)]	65 (27.5)	523 (35.3)	5.43	0.02
HAMD-17 score	32.1 ± 2.8	30.0 ± 2.9	10.56	< 0.001
HAMA score	24.2 ± 3.5	20.3 ± 3.1	17.78	< 0.001
BMI (kg/m^2)	24.7 ± 1.7	24.3 ± 2.0	2.62	0.009
Overweight [*n* (%)]	107 (45.3)	572 (38.6)	3.87	0.049

HAMD-17 17-item Hamilton Rating Scale for Depression, *HAMA* Hamilton Anxiety Rating Scale, *BMI* body mass index, *SA* suicide attempts

Table S2 Demographic and clinical characteristics in SA and non-SA patients categorized by sex

Variable	Male (*n* = 588)		Female (*n* = 1130)		*F* (*P*)		
	SA (*n* = 65)	Non-SA (*n* = 523)	SA (*n* = 171)	Non-SA (*n* = 959)	Sex	SA	Diagnosis × Sex
Age (year)	34.5 ± 13.0	33.0 ± 12.1	37.2 ± 12.4	35.5 ± 12.5	7.58 (0.006)	2.90 (0.09)	0.007 (0.93)
Age of onset (year) [a]	34.3 ± 13.0	32.8 ± 12.0	37.1 ± 12.2	35.3 ± 12.3	2.34 (0.13)	0.04 (0.85)	0.10 (0.32)
HAMD-17 score [b]	31.8 ± 3.0	30.1 ± 2.9	32.2 ± 2.7	30.0 ± 2.9	0.46 (0.50)	76.19 (< 0.001)	1.93 (0.17)
HAMA score [b]	23.9 ± 3.4	20.3 ± 3.2	24.4 ± 3.6	20.3 ± 3.1	0.86 (0.36)	237.17 (< 0.001)	1.16 (0.28)
BMI (kg/m^2) [b]	24.4 ± 1.8	24.4 ± 2.1	24.8 ± 1.7 *	24.3 ± 1.9 *	0.83 (0.36)	2.29 (0.13)	3.81 (0.05)
Overweight [*n* (%)]	24 (36.9)	215 (41.1)	83 (48.5) *	357 (37.2) *	-	-	-

[a]Adjusted for age. [b]Adjusted for age and onset of age. *Indicates significant difference ($P < 0.05$) between female SA and non-SA. *HAMD-17* 17-item Hamilton Rating Scale for Depression, *HAMA* Hamilton Anxiety Rating Scale, *BMI* body mass index, *SA* suicide attempts

Table S3 Association between each variable and SA in major depressive disorder (MDD) by categorized by sex using univariate logistic regression

**Variable**	**Male**				**Female**			
	β	***P***	***OR***	**95% CI**	***β***	***P***	***OR***	**95% CI**
Age	0.01	0.333	1.010	0.99 − 1.032	0.011	0.102	1.011	0.998 − 1.024
Age of onset	0.01	0.341	1.010	0.989 − 1.032	0.011	0.095	1.011	0.998 − 1.025
HAMD-17 score	0.186	< 0.0001	1.205	1.104 − 1.315	0.286	< 0.0001	1.331	1.250 − 1.418
HAMA score	0.300	< 0.0001	1.35	1.244 − 1.464	0.351	< 0.0001	1.421	1.343 − 1.502
BMI	-0.01	0.881	0.990	0.872 − 1.125	0.154	0.001	1.167	1.065 − 1.277
Overweight	-0.187	0.487	0.829	0.490 − 1.405	0.455	0.006	1.577	1.139 − 2.183

HAMD-17 17-item Hamilton Rating Scale for Depression, *HAMA* Hamilton Anxiety Rating Scale, *BMI* body mass index, *SA* suicide attempts

Table S4 Association between BMI and SA in major depressive disorder (MDD) by categorized by sex using multivariate logistic regression

**Variable**	**Male**				**Female**			
	β	***P***	***OR***	**95% CI**	***β***	***P***	***OR***	**95% CI**
HAMD-17 score	-0.080	0.19	0.924	0.820 − 1.040	0.004	0.326	1.041	0.961 − 1.128
HAMA score	0.349	< 0.0001	1.418	1.269 − 1.583	0.328	< 0.0001	1.388	1.296 − 1.487
BMI	-0.024	0.744	0.977	0.848- − 1.125	0.146	0.005	1.157	1.045 − 1.281

HAMD-17 17-item Hamilton Rating Scale for Depression, *HAMA* Hamilton Anxiety Rating Scale, *BMI* body mass index, *SA* suicide attempts

Table S5 Association between overweight and SA in major depressive disorder (MDD) by categorized by sex using multivariate logistic regression

**Variable**	**Male**				**Female**			
	β	***P***	***OR***	**95% CI**	***β***	***P***	***OR***	**95% CI**
HAMD-17 score	-0.080	0.186	0.923	0.820 − 1.039	0.038	0.351	1.039	0.959 − 1.125
HAMA score	0.349	< 0.0001	1.417	1.269 − 1.583	0.331	< 0.0001	1.392	1.300 − 1.491
Overweight	-0.179	0.538	0.836	0.473 − 1.478	0.449	0.016	1.566	1.087 − 2.257

HAMD-17 17-item Hamilton Rating Scale for Depression, *HAMA* Hamilton Anxiety Rating Scale, *SA* suicide attempts
